# Surgical Management of Acetabular Fractures: A Case Series

**DOI:** 10.5812/traumamon.7164

**Published:** 2013-05-26

**Authors:** Hassan Rahimi, Mohammad Gharahdaghi, Ali Parsa, Maryam Assadian

**Affiliations:** 1 Department of Orthopedic Surgery, Mashad University of Medical Sciences, Mashad, IR Iran; 2 Department of Orthopedic Surgery, Zahedan University of Medical Sciences, Zahedan, IR Iran

**Keywords:** Acetabulum, Surgical Procedures, Operative, Fractures, Bone

## Abstract

**Introduction:**

For decades, acetabular fractures were treated conservatively. Judet et al. in 1960s established the operative treatment of these fractures by continuous improvement of pre-operative evaluation and classification of fractures. Several studies demonstrated that accurate fracture reduction decreases the incidence of post-traumatic arthritis and improves functional outcome.

**Case Series:**

We report 67 consecutive patients who underwent surgical treatment for acetabular fracture; 44 patients were available for follow-up. In 35 (79.5%) cases, congruent reductions were achieved. The final mean Harris hip score was 81.8 (53-95). Functional outcomes according to Harris score were excellent and good in 31 patients (70.5%).

**Conclusions:**

The results of internal fixation of displaced acetabular fractures in our series were satisfactory.

## 1. Introduction

Fractures of acetabulum and pelvis constitute only 2% of all fractures (1, 2) but they are associated with significant morbidity and mortality due to associated injuries (3). Several studies demonstrated that accurate reduction and rigid internal fixation can decrease the incidence of post-traumatic arthritis and improve functional outcome (4-6). Clinical outcome after acetabular fracture surgery is difficult to predict. Poor bone stock in older patients, comminuted articular surface fractures and poly-trauma patients with multiple co-morbidities are adverse factors influencing outcome (7). Current trends in the treatment of these fractures include open reduction and internal fixation (8) according to the principles that apply to all inta-articular injuries. Judet et al. in 1960s classified these fractures and established the principles of operative management (9). Kebaish et al. in a long term follow–up study, indicated that the results of non-operative treatment in displaced acetabular fractures were inferior compared to those of operative treatment (30% satisfactory results versus 80% satisfactory results in the surgical group) (10). The current study reports 15-yr. results (1996-2010) and functional outcome of surgical treatment of variable types of displaced acetabular fractures.

## 2. Case Series

Between March 1996 and September 2010, 67 consecutive patients underwent surgical treatment for acetabular fractures at the Mashhad University Trauma Center. Surgical criteria were acetabular fractures with 2 mm or more displacement in the dome area of the acetabulum, roof arc angle measurement of less than 45 degrees, presence of intra-articular fragments, posterior joint instability, irreducible fracture/dislocations and the need to reconstruct the socket for total hip replacement.Unfortunately 4 cases died due to poor general health and associated injuries and 19 cases were lost to follow-up. Therefore there were 44 patients with complete data. Among these cases there were 34 male and 10 female patients; their age at the time of surgery was between 17 and 78 (mean 43.1 years old), in 19 cases the left acetabulum and in 25 cases the right acetabulum was fractured. The mechanism of injury was road-traffic accidents in 38 patients, fall from a height in 4 and in 2 cases the fractures were due to other causes.There were 9 associated injuries, including 2 cases of head injury,2 abdominal injuries, 2 incomplete sciatic nerve injuries (both had only proneal nerve involvement), one ipsilateral subtrochanteric fracture, one ipsilateral femoral shaft fracture and one contralateral femoral shaft fracture.After clinical evaluation and initial resuscitation, all patients had preoperative X-rays taken and a CT scan was also performed for patients admitted after 2001. All fractures were classified according to the criteria of Judet et al.; 16 were simple and 28 complex fractures (Table 1).

**Table 1.** Types of Fractures

Fracture type	Patients
**Simple**	
Anterior Wall	2
Anterior Column	0
Posterior Wall	11
Posterior Column	0
Transverse	3
**Complex**	
Both Column	15
Posterior Wall + Posterior Column	8
Anterior Wall + Anterior Column	0
Anterior Column + Posterior Hemitransverse	3
Posterior Wall + Transverse	2

Surgery was performed via the "Kocher-Langenbeck" (lateral position), "ilioinguinal" and "extended iliofemoral" approaches (11). In 7 cases trochantric osteotomy was performed for better reduction. Most of the patients were mobilized non-weight bearing 2 days after surgery; weight bearing progressively increased in the following 6 weeks with full weight bearing permitted after union of the fractures. Indomethacin was prescribed 75 mg daily for 6 weeks as a prophylaxis for heterotopic ossification. For prevention of DVT (from 2001 onward) low molecular-weight heparin was administered for 10 - 14 days post-operatively. Patients were followed up by clinical and radiological evaluation at 6 and 12 weeks, 6 months post-operatively and then yearly. Follow-up consisted of imaging evaluation and functional evaluation (Harris hip score). The average follow-up period was 62.1 months (range 13 - 156 months).

## 3. Conclusions

**Figure 1.**
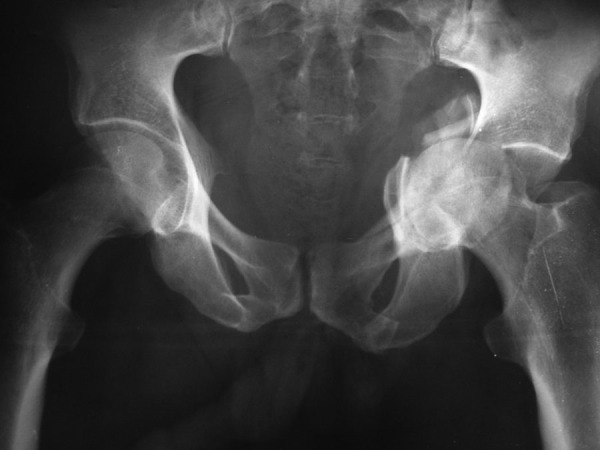
Comminuted Central Fracture-Dislocation

Thirty-six (81%) of patients were operated within 2 weeks of injury. Five cases were referred late to trauma center and 3 patients had associated injuries, thus 8 patients were operated after the ideal 2- week post-injury period. A Kocher-Langenbeck approach was used in 20 (45.4%) cases, an anterior ilioinguinal approach in 16 patients (36.4%), and an extended iliofemoral approach in 8 patients (18.2%).There was no residual instability or nonunion at the final follow-up. In 35 (79.5%) cases, congruent reductions were achieved. The remaining 9 patients had incongruent reduction of their fractures: In 5 patients both column fractures were operated 2 weeks following the injury, there were 3 central fracture dislocations with severe comminution (Figure 1) and 1 anterior column posterior hemi-transverse fracture. The final mean Harris hip score was 81.8 (53-95). Functional outcomes according to Harris score were excellent or good in 31 patients (70.5%) (Table 2), poor in 5 patients (11.4%) with mean Harris score = 59, all of which had incongruent reduction.

**Table 2.** Final Outcome Based on Harris Score After Surgery

Functional Outcome (Harris Hip Score)	Patients (%)
**Excellent**	11 (25 )
**Good**	20 (45.5)
**Fair**	8 (15.1)
**Poor**	5 (11.4)

**Figure 2.**
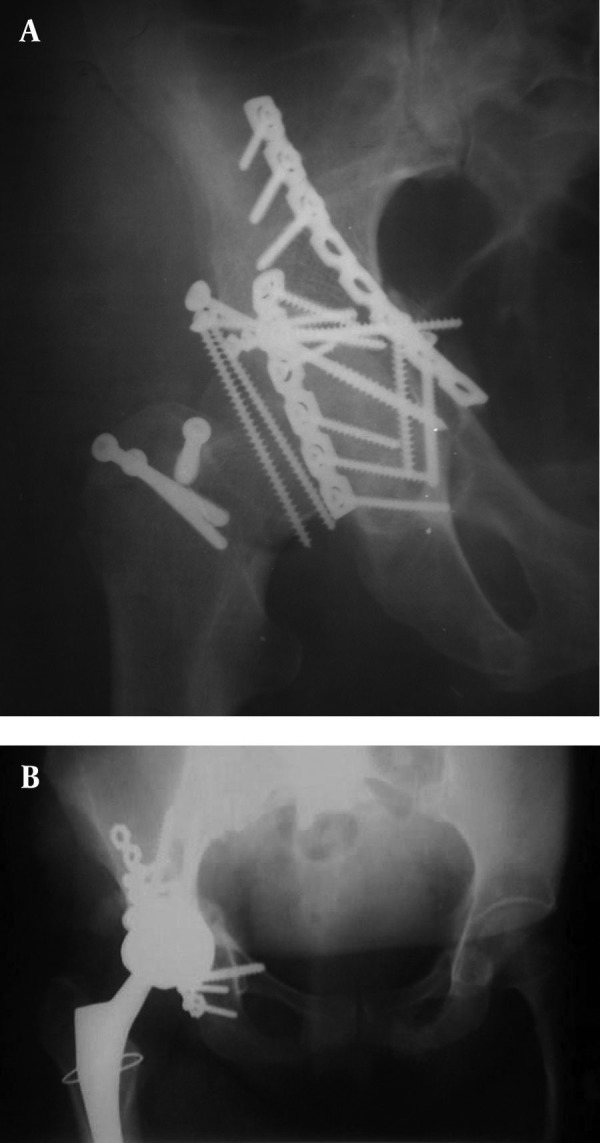
Secondary Hip Arthritis. A) Symptomatic hip arthritis after surgery; B) After total hip replacement

In the current series, radiographic congruency correlated well with functional scoring (P < 0.05, t-test). Mean Harris score of "incongruent reduction group" was 67.3 compared to 85.5 in the "congruent reduction group." It seems that, age affected functional results. Mean Harris score in patients under 40 was 85.3 compared to 79.7 in patients over 40 years old (P < 0/05, t-test).There were five (11.4%) early complications, including 2 (4.6%) deep vein thromboses treated by cardiologists and 3 (6.8%) wound infections treated by surgical debridement. There were 2 (4.6%) heterotopic ossifications (HO) following a Kocher-Langenbeck approach both of which were treated non-operatively and 2 (4.6%) patients developed symptomatic post-traumatic arthritis managed by total hip arthroplasty (Figures 2A and 2B). Reduction was lost in one of the patients after surgery ; there were no cases of implant failure at final follow-up.

Acetabular fractures are commonly classified by the "Letournel" and "Judet" system (1). This classification system describes the fracture in terms of elementary fractures and associated fractures (7). The outcome is potentially dependent on personal characteristics of the patient and circumstances of the accident (12). Type of fracture, displacement and comminution as well as concomitant diseases have been said to affect clinical outcome (13). In the current study, 70.5% of patients had good or excellent results which is comparable to several studies (11, 14, 15). Young patients aged < 40 years at the time of injury had better final outcomes; this correlation was also noted by Libergall et al. (16). It was shown that congruent reduction leads to much better functional outcome with statistically significant differences in Harris scores. Results of the current study indicated that the functional results correlate well with the quality of reduction. Non-congruent reduction was shown to be the main cause of unsatisfactory results. Extensile approaches to the hip joint have a high rate of complications (17-19); Up to 67% develop heterotopic ossification (HO) even with the use of post-operative irradiation as prophylaxis after the extensile approach (16, 20). Infection rates reported in such studies vary from 0-3% (16) to 5-12% (16, 19); and the infection rate in the current study was 6.8% which is comparable with these studies. Thromboembolic complications had a low rate of 4.6% comparable with the existing reported cases although MRV (Magnetic ResonanceVenography) (21) was not used in the current study. Two (4.5%) patients went on to have total hip replacement within 2 years of surgery, this incidence was low in contrast to other studies (22, 23). It should be mentioned that 88.6% of the patients in the current study underwent the operation within 2 weeks after injury. In conclusion, a good/excellent functional and radiological outcome was observed in 70.5% of operatively-treated patients. A significant association was found between a sub-optimal clinical outcome after fracture of the acetabulum and 3 prognostic factors (imperfect fracture reduction, complex fractures and age). Complex fractures raised the odds ratio of sub-optimal to satisfactory outcome at least 4.6 times compared to a simple fracture. Results of the current study are comparable with other studies (12, 15, 16).

## References

[1] Hesp WL, Goris RJ (1988). Conservative treatment of fractures of the acetabulum. Results after longtime follow-up.. Acta Chir Belg..

[2] Ragnarsson B, Jacobsson B (1992). Epidemiology of pelvic fractures in a Swedish county.. Acta Orthop Scand..

[3] Van Veen IHPAA, Van Leeuwen AAM, Van Popta T, Van Luyt PA, Bode PJ, Van Vugt AB (1995). Unstable pelvic fractures: a retrospective analysis.. Injury..

[4] Matta JM, Anderson LM, Epstein H, Hendricks P (1986). Fractures of the acetabulum. A retrospective analysis.. Clin Orthop Relat Res..

[5] Matta JM, Mehne DK, Roffi R (1986). Fractures of the acetabulum. Early results of a prospective study.. Clin Orthop Relat Res..

[6] Ragnarsson B, Mjoberg B (1992). Arthrosis after surgically treated acetabular fractures. A retrospective study of 60 cases.. Acta Orthop Scand..

[7] McMaster J, Powell J (2005). Acetabular fractures.. Curr Orthop..

[8] Kumar A, Shah NA, Kershaw SA, Clayson AD (2005). Operative management of acetabular fractures. A review of 73 fractures.. Injury..

[9] Judet R, Judet J, Letournel E (1964). [Fractures of the Acetabulum].. Acta Orthop Belg..

[10] Chiu FY, Chen CM, Lo WH (2000). Surgical treatment of displaced acetabular fractures - 72 cases followed for 10 (6-14) years.. Injury..

[11] Kebaish AS, Roy A, Rennie W (1991). Displaced acetabular fractures: long-term follow-up.. J trauma..

[12] Murphy D, Kaliszer M, Rice J, McElwain JP (2003). Outcome after acetabular fracture. Prognostic factors and their inter-relationships.. Injury..

[13] Ovre S, Madsen JE, Roise O (2008). Acetabular fracture displacement, roof arc angles and 2 years outcome.. Injury..

[14] Matta JM (1996). Fractures of the acetabulum: accuracy of reduction and clinical results in patients managed operatively within three weeks after the injury.. J Bone Joint Surg Am..

[15] Deo SD, Tavares SP, Pandey RK, El-Saied G, Willett KM, Worlock PH (2001). Operative management of acetabular fractures in Oxford.. Injury..

[16] Liebergall M, Mosheiff R, Low J, Goldvirt M, Matan Y, Segal D (1999). Acetabular fractures. Clinical outcome of surgical treatment.. Clin Orthop Relat Res..

[17] Alonso JE, Davila R, Bradley E (1994). Extended iliofemoral versus triradiate approaches in management of associated acetabular fractures.. Clin Orthop Relat Res..

[18] Moroni A, Caja VL, Sabato C, Zinghi G (1995). Surgical treatment of both-column fractures by staged combined ilioinguinal and Kocher-Langenbeck approaches.. Injury..

[19] Starr AJ, Watson JT, Reinert CM, Jones AL, Whitlock S, Griffin DR (2002). Complications following the "T extensile" approach: a modified extensile approach for acetabular fracture surgery-report of forty-three patients.. J Orthop Trauma..

[20] Kaempffe FA, Bone LB, Border JR (1991). Open reduction and internal fixation of acetabular fractures: heterotopic ossification and other complications of treatment.. J Orthop Trauma..

[21] Stannard JP, Lopez-Ben RR, Volgas DA, Anderson ER, Busbee M, Karr DK (2006). Prophylaxis against deep-vein thrombosis following trauma: a prospective, randomized comparison of mechanical and pharmacologic prophylaxis.. J Bone Joint Surg Am..

[22] Catalano JB, Born CT (1997). Total hip arthroplasty after acetabular fracture treated initially with open reduction and internal fixation.. Oper Tech Orthop..

[23] Ranawat A, Zelken J, Helfet D, Buly R (2009). Total hip arthroplasty for posttraumatic arthritis after acetabular fracture.. J Arthroplasty..

